# Synonymous Codon Usages as an Evolutionary Dynamic for *Chlamydiaceae*

**DOI:** 10.3390/ijms19124010

**Published:** 2018-12-12

**Authors:** Zhaocai Li, Wen Hu, Xiaoan Cao, Ping Liu, Youjun Shang, Jizhang Zhou

**Affiliations:** 1 State Key Laboratory of Veterinary Etiological Biology, Lanzhou Veterinary Research Institute, Chinese Academy of Agricultural Sciences, Lanzhou 730046, China; lizhaocai@caas.cn (Z.L.); huwen_only@163.com (W.H.); caoxiaoan@caas.cn (X.C.); liuping_44@163.com (P.L); 2 Gansu Police Vocational College, Lanzhou 730046, China; 3 Biomedical Research Center, Northwest Minzu University, Lanzhou 730030, China

**Keywords:** *Chlamydia*, synonymous codon usage bias, amino acid usage bias, information entropy, evolutionary dynamic

## Abstract

The family of *Chlamydiaceae* contains a group of obligate intracellular bacteria that can infect a wide range of hosts. The evolutionary trend of members in this family is a hot topic, which benefits our understanding of the cross-infection of these pathogens. In this study, 14 whole genomes of 12 *Chlamydia* species were used to investigate the nucleotide, codon, and amino acid usage bias by synonymous codon usage value and information entropy method. The results showed that all the studied *Chlamydia* spp. had A/T rich genes with over-represented A or T at the third positions and G or C under-represented at these positions, suggesting that nucleotide usages influenced synonymous codon usages. The overall codon usage trend from synonymous codon usage variations divides the *Chlamydia* spp. into four separate clusters, while amino acid usage divides the *Chlamydia* spp. into two clusters with some exceptions, which reflected the genetic diversity of the *Chlamydiaceae* family members. The overall codon usage pattern represented by the effective number of codons (ENC) was significantly positively correlated to gene GC3 content. A negative correlation exists between ENC and the codon adaptation index for some *Chlamydia* species. These results suggested that mutation pressure caused by nucleotide composition constraint played an important role in shaping synonymous codon usage patterns. Furthermore, codon usage of *T3ss* and *Pmps* gene families adapted to that of the corresponding genome. Taken together, analyses help our understanding of evolutionary interactions between nucleotide, synonymous codon, and amino acid usages in genes of *Chlamydiaceae* family members.

## 1. Introduction

*Chlamydia* spp. are a group of obligate intracellular bacteria that are widely distributed throughout the world, causing a variety of diseases in humans and animals [1]. To date, 12 species have been identified in the single genus of the family *Chlamydiaceae*: *C. trachomatis*, *C. pneumoniae*, *C. muridarum*, *C. suis*, *C. psittaci*, *C. pecorum*, *C. abortus*, *C. felis*, *C. caviae*, *C. avium*, *C. gallinacea*, and *C. ibidis* [2,3]. Among these species, *C. trachomatis* and *C. pneumonia* mainly cause diseases in humans, while other species often cause animal diseases and most have zoonotic potential [4]. *C. trachomatis* infects the ocular and genital mucosa. It is the leading cause of infectious preventable blindness in developing countries and contributes to the most prevalent bacterial sexually transmitted diseases (STDs) throughout the world [5,6]. Urogenital infection of *C. trachomatis* may cause serious sequelae including pelvic inflammatory disease (PID), infertility, and ectopic pregnancy [6]. The mouse pathogen *C. muridarum* is usually used as a model for understanding of *C. trachomatis* genital tract infections [7]. *C. pneumoniae* is an important respiratory pathogen that causes approximately 5% of all cases of bronchitis and is believed to be responsible for about 10% of community-acquired pneumonia cases [8]. *C. psittaci* has widespread occurrence in poultry and wild birds causing psittacosis or ornithosis or developing non-specific symptoms and has undisputable zoonotic character, causing severe flu-like infections in humans [9]. Infection with other chlamydial species also affects mucosal membranes, leading to fertility disorders, severe conjunctivitis, or pneumonia. For instance, *C. abortus* is the causative agent of abortion in sheep, goat, cattle, pig, and other mammals [10], and *C. pecorum* is known to cause disorders of the intestinal and genital tracts, as well as arthritis, in ruminants and pigs [11]. *C. suis* is found to be the most prevalent chlamydial species in pigs while *C. felis* and *C. caviae* are distinguished by their high host specificity having adapted to cats or guinea pigs, respectively. *C. avium*, *C. gallinacea*, and *C. ibidis* are newly identified chlamydial species from birds with unclear pathogenicity [3,12,13].

All the *Chlamydia* spp. share a common but unique biphasic developmental cycle, involving an infectious, but metabolically inactive elementary body (EB), which invades host cells, and a noninfectious metabolically active reticulate body (RB), which resides and multiplies within an intracellular non-fusogenic vacuole-like cytoplasmic inclusion [14]. Although many aspects of virulence and pathogenic mechanisms for most species are not clear yet, several common toxic factors have been identified. For instance, the polymorphic membrane proteins (Pmps) of *Chlamydia* are thought to be involved in the process of chlamydial adhesion, tissue tropism, and immune responses induction [15,16,17]. Type III secretion system plays a vital role in the formation and development of chlamydial inclusion by delivering effector proteins into their target host cells [18,19]. The effectors interfere with diverse host cellular processes including signaling, cytoskeletal rearrangements, and vesicle trafficking to enhance bacterial entry, establish a replicative niche and evade innate immunity [20,21].

As obligate intracellular microorganisms, co-evolution between *Chlamydiae* and their hosts enables them to adapt for extracellular infectious and intracellular reproductive life cycle to their host. These are closely related to the pathogenicity of *Chlamydiae* [22]. With the development of sequencing technology, complete genomes of members of *Chlamydiaceae* are available for investigating their pathogenic mechanisms as well as phyletic evolution. *Chlamydia* spp. like other endocellular bacteria such as *Rickettsia* spp., have substantially reduced, A/T rich genomes (1.04 Mb with 58.7% of A+T, encoding 895 open reading frames for *C. trachomatis*) [23,24]. The lack of many metabolic enzymes makes them reliant on the hosts for many of their metabolic requirements [25]. It is evident that the endocellular lifestyle is the base of pathogenicity of these bacteria. Study on the evolutionary strategy of genomes may supply some information about their pathogenic mechanisms. The genetic information about interplay between nucleotide, synonymous codon, and amino acid usages could reflect evolutionary dynamics of *Chlamydia* spp. However, evolutionary drivers proper to *Chlamydia* themselves at nucleotide and amino acid usage levels have not been investigated yet. Nucleotide usage variation was considered as an important evolutionary dynamic, however, synonymous codon usages were able to minimize error impacts by increasing tolerance for some point mutations to stabilize amino acid usages [26,27,28,29,30]. Investigations of synonymous codon usage identified several evolutionary dynamics which impact overall codon usage patterns, including natural/translation selection, mutation pressure, hydrophobicity and hydrophilicity of the protein, protein folding, and host preferences [31,32,33,34,35,36]. Depending on the information about nucleotide, synonymous codon, and amino acid usage from the available genomes of *Chlamydia* spp., we tried to identify the evolutionary dynamics in shaping the unique genetic features of these bacteria in the family of *Chlamydiaceae*.

## 2. Results

### 2.1. Bias A/T versus G/C in Chlamydia *spp.* Genes

To better clarify the organization of nucleotide usages at gene levels of *Chlamydia* spp., the nucleotide contents of genes in each species were calculated. The average contents of the four nucleotides represented similar patterns in the 12 species, namely bias for high AT content versus low GC content (Table 1). Furthermore, nucleotide usages at third codon positions strongly influenced the organization at gene levels (Table 1). Generally, the overall nucleotide usages at gene levels had an obvious effect on the organization of nucleotide usages at third codon positions, suggesting synonymous codon usages could be influenced by the stable organization of nucleotide usages at gene levels of *Chlamydia* spp.

### 2.2. Nucleotide Usage Bias at Gene Levels of Chlamydia *spp.*

**Figure 1:**
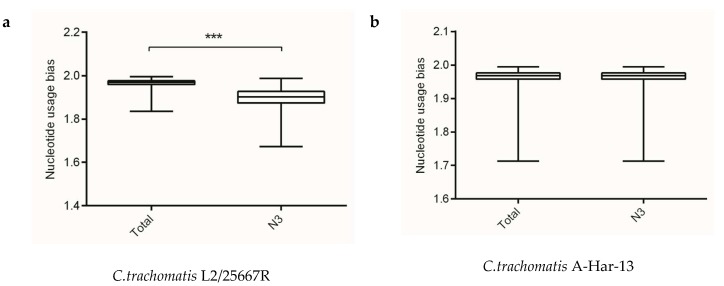

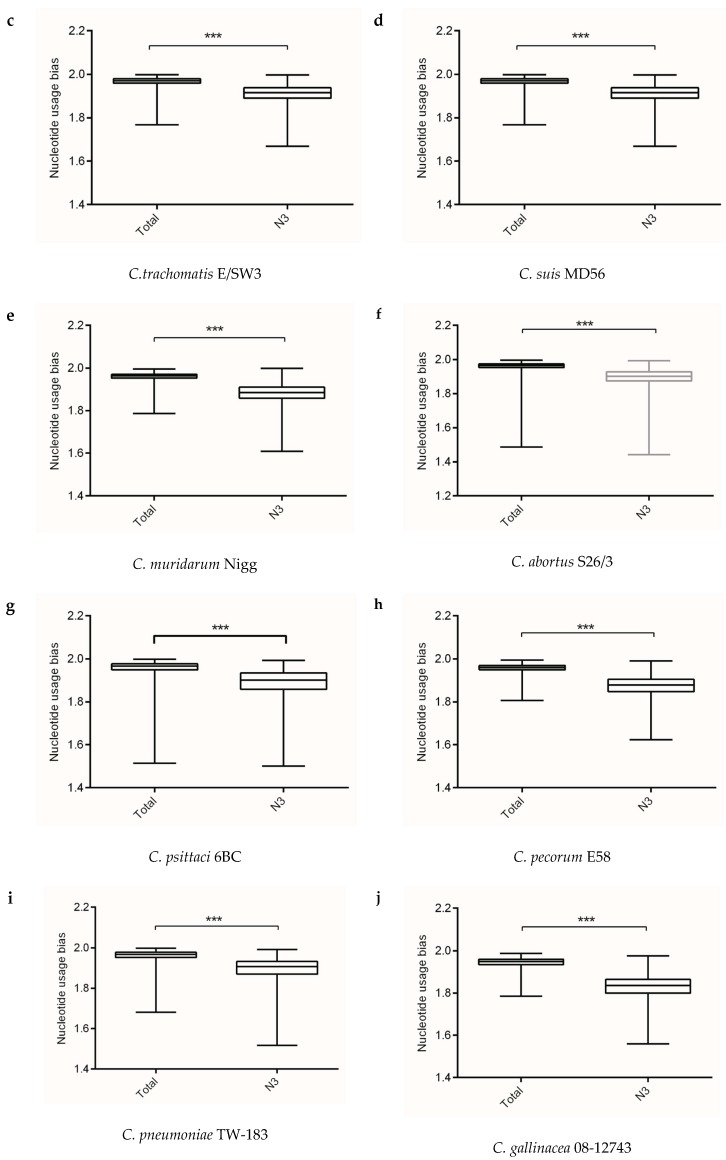

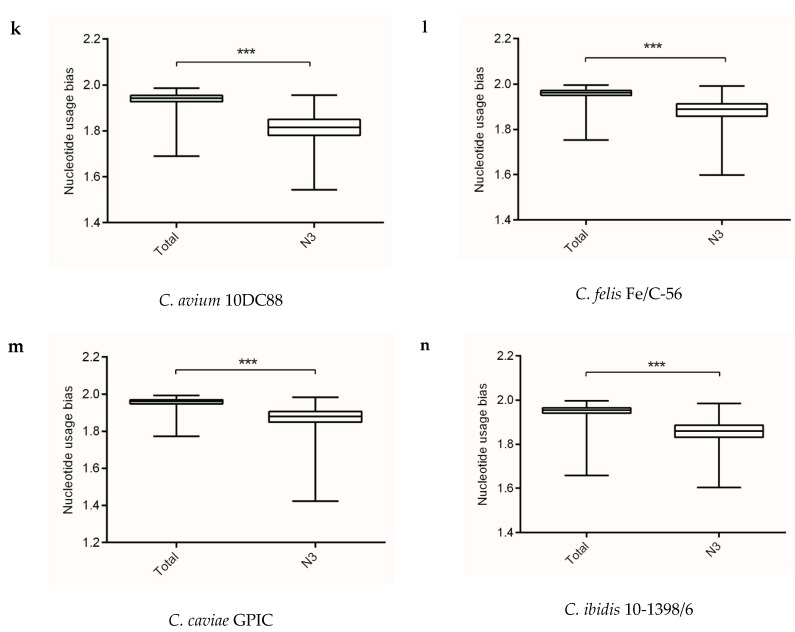
The overall codon usage bias and codon usage bias at the third codon position at gene levels of the *Chlamydia* spp. (**a**) *C. trachomatis* L2/25567R, (**b**) *C. trachomatis* A-HAR-13, (**c**) *C. trachomatis* E/SW3, (**d**) *C. suis* MD56, (**e**) *C. muridarum* Nigg, (**f**) *C. abortus* S26/3, (**g**) *C. psittaci* 6BC, (**h**) *C. pecorum* E58, (**i**) *C. pneumoniae* TW-183, (**j**) *C. gallinacea* 08-12743, (**k**) *C. avium* 10DC88, (**l**) *C. felis* Fe/C-56, (**m**) *C. caviae* GPIC, (**n**) *C. ibidis* 10-1398/6. *** *p* < 0.001.

Analysis of information entropy showed an overall nucleotide usage bias derived from the four nucleotide contents (Table 1). Figure 1 showed that extents of nucleotide usage bias at third codon positions was generally stronger than the overall nucleotide usage bias in genes of *Chlamydia* spp. This result implied that mutation pressure caused by nucleotide composition played an important role in shaping synonymous codon usages in *Chlamydia* spp. In addition, the three biovars of *C. trachomatis* exhibited different nucleotide usage biases at third codon positions, with *C. trachomatis* lymphogranuloma venereum biovar strain L2/25567R significantly different from *C. trachomatis* trachoma biovar strain A-HAR-13 and *C. trachomatis* genital tract infection biovar strain E/SW3 (Figure 1). This result might suggest that mutation pressure functioned as a regulator for different biovars in the *C. trachomatis*.

### 2.3. Nucleotide Usage Influencing Codon Usage

We quantified bias in synonymous codon usage using relative synonymous codon usage (RSCU). All over-represented synonymous codons ended with A or T, while all under-represented synonymous codons ended with G or C (Table S2). These genetic features strongly reflected obvious constraints on nucleotide composition shaping synonymous codon usage biases in *Chlamydia*. Note that despite the dominating evolutionary dynamic of nucleotide usage patterns on codon usages and their variation in *Chlamydia*, some synonymous codons usage patterns showed that constraints on nucleotide composition were not the only constraints affecting the evolutionary dynamics of *Chlamydia* gene contents. In Table S2, although the four dominant synonymous codons (CTA for Leu, ATA for Ile, AGT for Ser and CCA for Pro) ended with A or T, corresponding RSCU values were less than 1.0 in the 12 species, suggesting that usage of the four synonymous codons was suppressed. Similar genetic features were also found in stop codons usages. In Table S3, despite bias for stop codon TAA in all 12 *Chlamydia*, stop codon TGA was less used than stop codon TAG, despite their identical nucleotide contents. These findings implied that translational constraints modified usages of specific synonymous codons and stop codons.

Compositional asymmetry of nucleotide contents between the replicational leading and lagging strands is common in bacteria [37] including *Chlamydiae*. To quantify the synonymous codon usage variations in the leading and lagging strand of each *Chlamydia* spp., we used PCA to reflect overall codon usage pattern. The results showed different codon usages of genes located on the leading and lagging strands for most of the *Chlamydia* spp. (Figure S1), which was consistent with the previous reports [38,39]. However, the codon usages in *C. avium* and *C. abortus* did not show separation between leading and lagging strands.

### 2.4. Genetic Diversity of Chlamydia *spp.* in Codon and Amino Acid Usage

**Figure 2:**
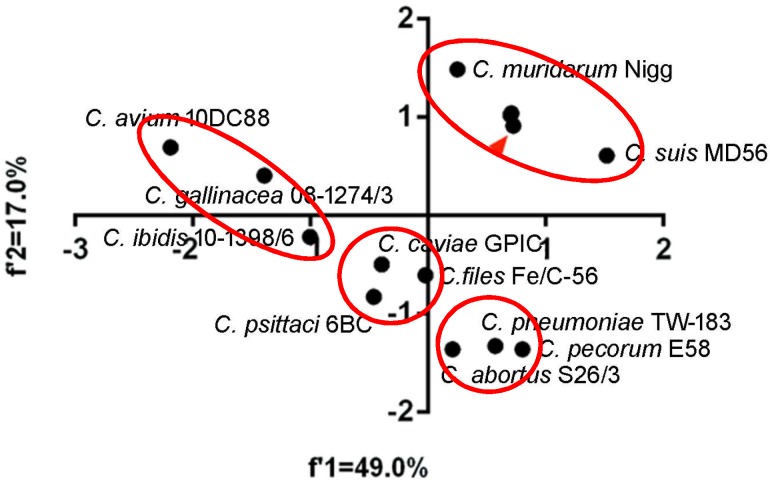
Principal component analysis of codon usage patterns at gene levels of the *Chlamydia* spp. The red triangle points to the three biovar strains of *C. trachomatis* (trachoma biovar strain A-HAR-13, genital tract infection biovar strain E/SW3, and lymphogranuloma venereum biovar strain L2/25567R), which highly overlap each other. The *Chlamydia* spp. clustered in separate groups were highlighted in red ellipses.

**Figure 3:**
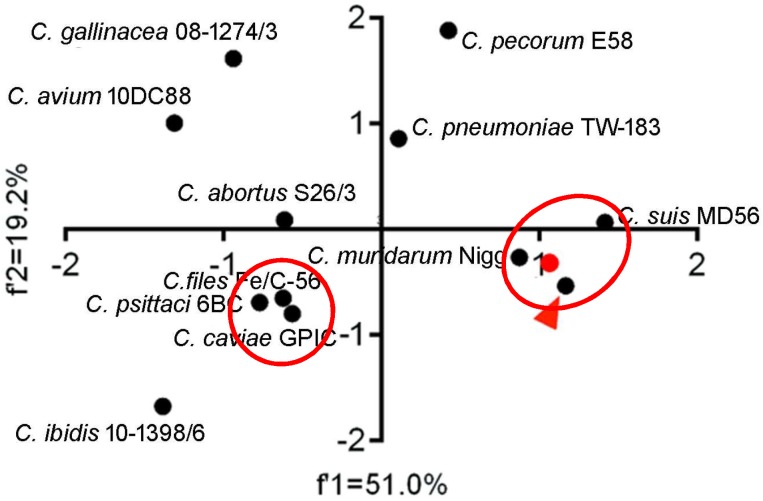
Principal component analysis of amino acid usage patterns of *Chlamydia* spp. The red triangle points to the two *C. trachomatis* strains (genital tract infection biovar strain E/SW3 and lymphogranuloma venereum biovar strain L2/25567R), which highly overlap each other, while the red shows the trachoma biovar strain A-HAR-13). The *Chlamydia* spp. clustered in separate groups were highlighted in red ellipses.

To quantify the overall codon usage trends from synonymous codon usage variations of *Chlamydia* spp., we used PCA to reflect overall codon usage trends. Generally, the first (f’1) and second (f’2) PCA axis accounted for 49.0% and 17.0% of the total codon usage variation, respectively. The three biovars of *C. trachomatis* almost perfectly overlapped and clustered with *C. suis* and *C. muridarum*, while *C. pecorum*, *C. pneumoniae* and *C. abortus* clustered together. *C. caviae*, *C. felis,* and *C. psittaci* formed a third cluster. Interestingly, the newly identified species, *C. avium*, *C. gallinacean* and *C. ibidis* showed different overall codon usage trends (Figure 2). The first (f’1) and second (f’2) PCA axis accounted for 51.0% and 19.2% of the total amino acid usage variation, respectively. The amino acid usage patterns for *C. trachomatis*, *C. muridarum*, *C. suis*, *C. felis*, *C. peittaci* and *C. caviae* could be divided into the two genetic clusters, however, the others owned their specific amino acid usage patterns (Figure 3). These results suggested that both codon usage and amino acid usage patterns could be regarded as evolutionary dynamics related to the balance between mutation pressure and natural selection for driving the evolution of the *Chlamydia* spp.

### 2.5. Multiple Selection Forces Influencing Codon Usage Patterns in Chlamydia *spp.*

**Figure 4:**
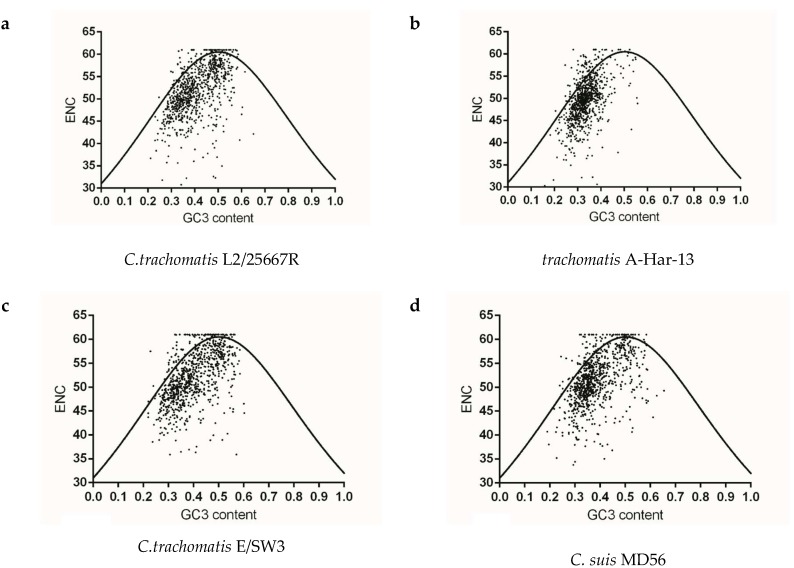

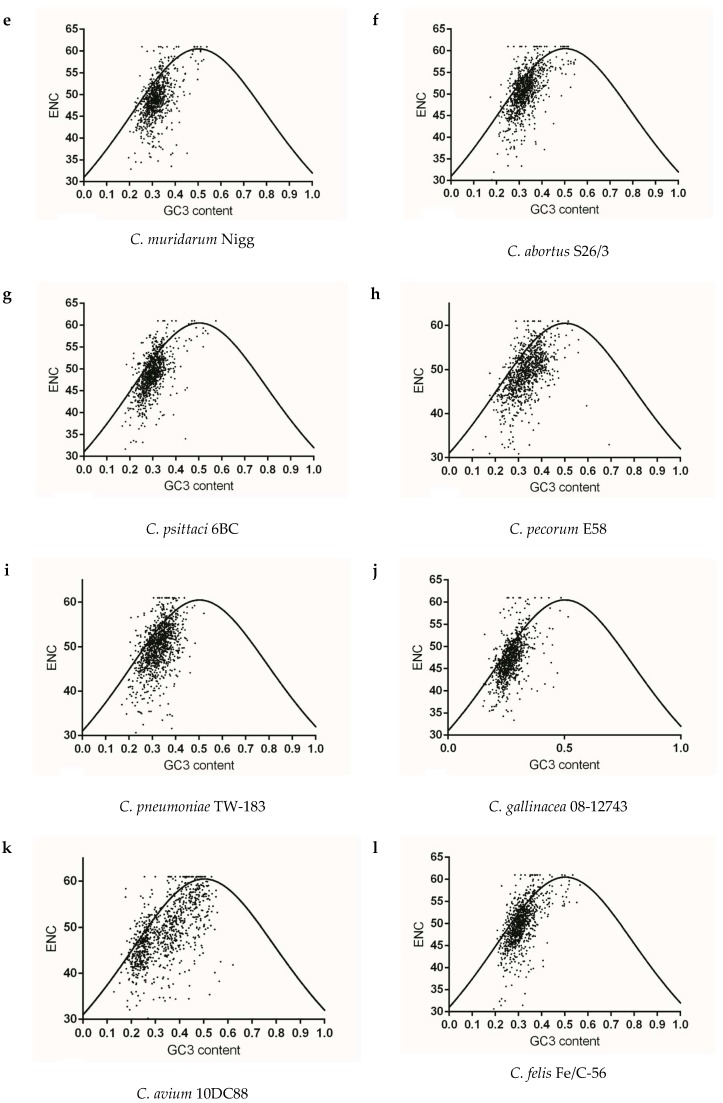

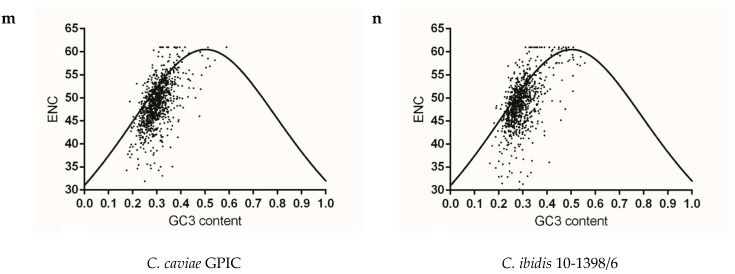
The relationship between the overall codon usage patterns represented by ENC values and GC composition at the third synonymous codon position (GC3) at gene levels of the *Chlamydia* spp. (**a**) *C. trachomatis* L2/25567R, (**b**) *C. trachomatis* A-HAR-13, (**c**) *C. trachomatis* E/SW3, (**d**) *C. suis* MD56, (**e**) *C. muridarum* Nigg, (**f**) *C. abortus* S26/3, (**g**) *C. psittaci* 6BC, (**h**) *C. pecorum* E58, (**i**) *C. pneumoniae* TW-183, (**j**) *C. gallinacea* 08-12743, (**k**) *C. avium* 10DC88, (**l**) *C. felis* Fe/C-56, (**m**) *C. caviae* GPIC, (**n**) *C. ibidis* 10-1398/6.

To identify whether gene codon usage patterns in each *Chlamydia* spp. were shaped solely by mutation pressure, natural selection, or both, ENC v.s GC3 content maps were constructed for each strain. The vast majority of plots for each species did not overlap the expected curve and were below this curve, and the below-curve scattering plots reflected the dominating effects of natural selection on genes of each species. As for the closely related species *C. trachomatis, C. muridarum* and *C. suis*, codon usage pattern at gene levels of *C. trachomatis* A-HAR-13 and *C. muridarum* Nigg represented more limited codon usage patterns than those of *C. trachomatis* L2/25567R, *C. trachomatis* E/SW3 and *C. suis* MD56 (Figure 4a–e). For *C. pneumoniae* and most of the mammal infecting *Chlamydia* spp., similar codon usage patterns among the corresponding genome were observed (Figure 4f–i,l–m). While the three newly identified bird-infesting *Chlamydia* spp., codon usage patterns of *C. avium* 10DC88 were more limited than those of *C. gallinacea* and *C. ibidis* (Figure 4j,k,n). To better identify the role of mutation pressure from gene nucleotide composition, correlations between ENC and GC3 contents of gene were calculated. ENC and GC3 content are positively correlated in all species (Table 2), suggesting that mutation pressure in all *Chlamydia* has dominant roles in shaping codon usage. In addition, significant negative correlation with the relative rank (*r* value ranging from −0.308 to −0.067) was found between CAI and ENC for all strains, excluding *C. suis* MD56, *C. pecorum* E58 and *C. pneumoniae* TW-183 (Table 3), implying that the obvious effect of codon usage bias the on codon usage pattern of gene population was just one among several evolutionary dynamics, compared with the role of mutation pressure.

### 2.6. High Codon Usage Adaptation of T3ss and Pmps Gene Families to that of Corresponding Genome

**Figure 5:**
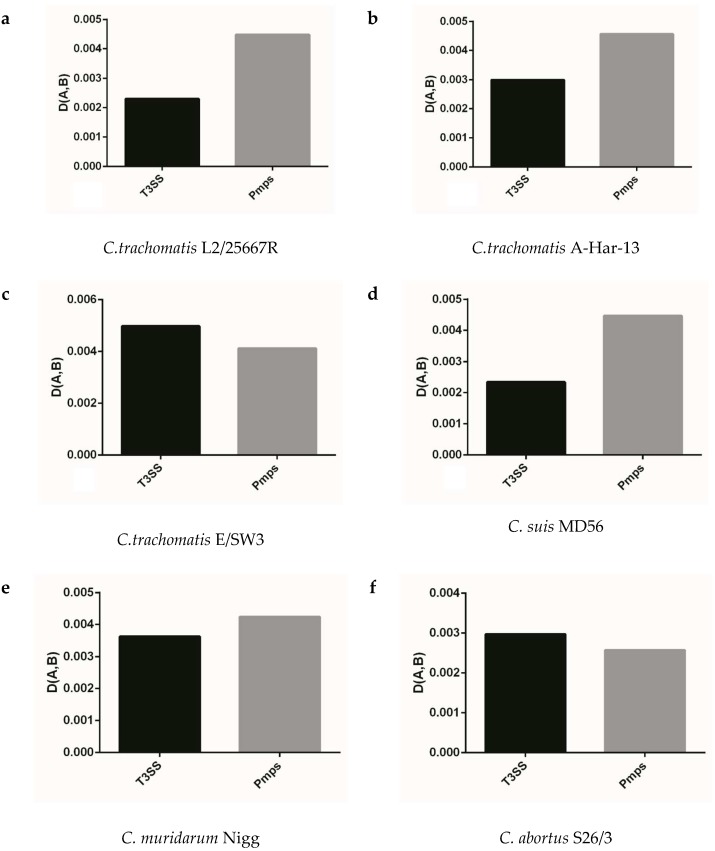

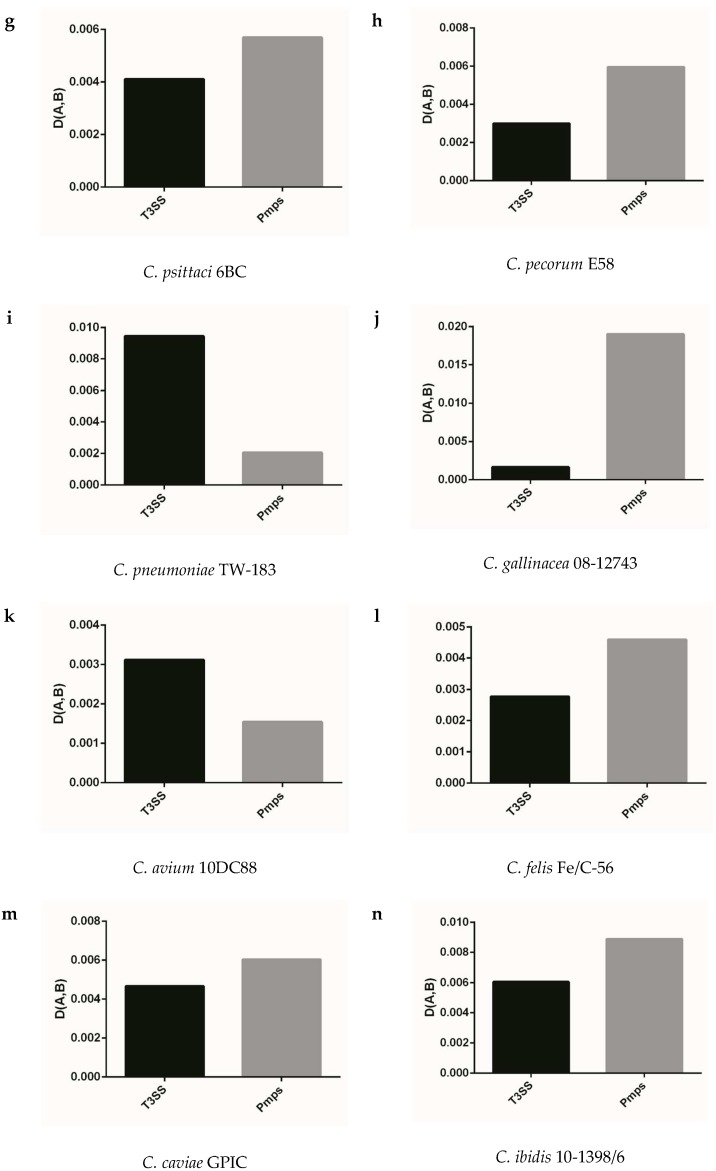
The adaptation of synonymous codon usage patterns between *T3ss* and *Pmps* gene families to the corresponding genome. (**a**) *C. trachomatis* L2/25567R, (**b**) *C. trachomatis* A-HAR-13, (**c**) *C. trachomatis* E/SW3, (**d**) *C. suis* MD56, (**e**) *C. muridarum* Nigg, (**f**) *C. abortus* S26/3, (**g**) *C. psittaci* 6BC, (**h**) *C. pecorum* E58, (**i**) *C. pneumoniae* TW-183, (**j**) *C. gallinacea* 08-12743, (**k**) *C. avium* 10DC88, (l) *C. felis* Fe/C-56, (**m**) *C. caviae* GPIC, (**n**) *C. ibidis* 10-1398/6.

We analyzed the extent of codon usage adaptation between *T3ss*, *Pmps* gene families and the corresponding genome to better identify the role of codon usage of gene population in the target gene, which played important roles in life cycle of *Chlamydia*. As shown in Figure 5, the two gene families generally had strong codon usage adaptation to the corresponding genome (*D (A,B)* < 0.1) and failed to follow the similar model of codon usage pattern in *Chlamydia* spp. In the closely related species of *C. trachomatis*, *C. muridarum* and *C. suis*, the *T3ss* and *Pmps* genes had a similar adaptation of codon usage in all strains excluding *C. suis* MD56 (Figure 5a–e). The *T3ss* and *Pmps* gene families had an obviously different adaptation of codon usage in *C. pneumoniae* TW-183 when compared with its closely related *Chlamydia* spp, such as *C. psittaci*, *C. pecorum*, *C. abortus*, *C. felis*, and *C. caviae* (Figure 5i,f,g,h,l,m). While for the three newly identified bird *Chlamydia* spp. (*C. gallinacean*, *C. avium* and *C. ibidis*), the two gene families had an obviously different adaptation of codon usage in *C. gallinacean* 08-1274/3, compared with the other two species (Figure 5j,k,n). Since *T3ss* and *Pmps* genes had specific adaptations of codon usage in the corresponding genome, the strong codon usage adaptation of the two gene families to the corresponding genome implied evolutionary dynamic derived from genome organization influencing codon usage pattern of genes, which played an important role in the life cycle of *Chlamydiae*.

## 3. Discussion

Here, we gave an evolutionary insight into the relationship between nucleotide usages and codon usages in genomes of *Chlamydiaceae* family members, by means of information entropy, RSCU, ENC, CAI and a similarity index of codon usage adaptation. Although mutation pressure derived from nucleotide usages was identified as the dominant evolutionary dynamic in genomes of *Chlamydia* spp., other evolutionary dynamics, such as natural selection, also influenced codon usage patterns to modify the evolutionary trends of *Chlamydia* spp. In previous reports, GC3 content was regarded as a ruler, which is often used for reflecting influences of the overall nucleotide composition variations on codon usages at gene levels [31,40,41,42,43]. The four nucleotide bases are regarded as footstones for genomic organization of microorganisms, the systemic and general estimation of four nucleotide usage patterns is better than the estimation of GC3 content or AT3 content when displaying the roles of nucleotide usage patterns in formation of synonymous codon usage patterns [44,45]. Members of *Chlamydiaeace* have similar genomes and share almost the same gene contents although they infect different hosts with pathogenic diversity [46]. Quantification of four nucleotide usage variations in gene population of the bacteria is a benefit for the overall nucleotide usage bias. A great deal of genetic information, such as the origin of *Chlamydia* spp., efficient nutrient usage, and the regulation/expression of genes in corresponding genomes, existed in the complex interplay between the four base (A, C, T and G) usage variations [47,48]. The information entropy method is able to systemically display this complex weight ratio between the four bases. The nucleotide usage bias at the gene levels of *Chlamydia* spp. reflects the role of efficient nutrient usage in strand-specific nucleotide usage and GC content. Besides, almost all bacterial genomes exhibit nucleotide compositional asymmetry between the replicational leading and lagging strands; therefore, there is an excess of nucleotides G relative to C in the leading strand and of C to G in the lagging strand [36]. This study, in accordance with previous studies, showed that the nucleotide compositional asymmetry contributed to the codon usage bias of genes located in leading and lagging strands in *Chlamydia* spp. [37,38], which showed the influence of nucleotide usage bias on codon usages. Interestingly, the codon usage patterns derived from genomes of *C. abortus* and *C. avium* displayed differences from other *Chlamydia* spp. in this study. It implied that gene location in the corresponding genome might serve as one evolutionary dynamic for codon usage formation in *Chlamydia* spp. Generally, AT-rich was found in gene levels of these genomes of *Chlamydia* spp., suggesting that AT-rich in *Chlamydia* spp. genomes could be an evolutionary feedback for their genome, losing many genes that were related to metabolic activities. AT-rich in gene levels of bacteria genome enabled the bacteria to replicate themselves with a small amount of energy [29,32,49,50,51].

The nucleotide usage bias at the third codon position was generally stronger than the overall nucleotide usage bias at the gene level in all *Chlamydia* spp., suggesting that the dominant evolutionary dynamic was caused by the nucleotide composition function on the codon usage pattern of *Chlamydiaceae*. Interestingly, the overall nucleotide usage bias, nucleotide usage bias at the third codon position and synonymous codon usage pattern represented similar patterns in *Chlamydiaceae*, respectively. Currently, there are two evolutionary theories that explain why genetic code changes do not result in extinction of the species: the ‘codon capture’ theory and the ‘ambiguous intermediate’ theory [52]. For instance, *Mycoplasma*, a group of extracellular bacteria, have synonymous codon usage patterns that are regulated by the ‘codon capture’ theory [53,54] due to their extreme nucleotide usage bias (AT-rich). On the other hand, *Chlamydia* spp., the obligate intercellular bacteria, which do not contain extreme nucleotide usage bias at gene levels should not be mediated by the ‘codon capture’ theory but might follow the ‘ambiguous intermediate’ theory.

As obligate intercellular bacteria, the development cycle of *Chlamydiae* includes a unique intracellular stage when the microorganisms undergo growth and proliferation in the chlamydial inclusions inside the host cytoplasm, whereby the living activities of *Chlamydiae* deeply interacts with the host cellular processes. The parasitic life-style of *Chlamydia* spp. has driven them to adapt to the harsh intracellular environment during the evolutionary process. It has been accepted that co-evolution between intercellular pathogens and hosts can be performed by positive selection from the immune response of hosts and mutation pressure from pathogens [55,56]. Approximately, two-thirds of predicted proteins are shared across *Chlamydia* spp., which reflects genetic conservation and the evolutionary constraints that are imposed by their intracellular lifestyle and conserved developmental cycle [25,57]. Similarly, the members of *Chlamydiaceae* undergo a significant genomic degradation in the evolutionary process when compared with *Parachlamydia acanthamoebae* UWE25, a symbiont of ubiquitous protozoa, which is considered the evolutionary homolog of the last common ancestor of *Chlamydia* spp. with a genome twice as large in size [39]. The investigation into evolutionary dynamics of this family would be a benefit for better understanding the genetic trends of *Chlamydiae*. Compared with genetic diversity caused by amino acid usage in *Chlamydia* spp., evolutionary divergence caused by codon usage can separate *Chlamydia* spp. into different subgroups, suggesting codon usages play an important role in evolutionary trends in the *Chlamydiaceae* family. Horizontal gene transfer in *Chlamydia* plays an important role in sustaining a wide range of susceptible hosts [58]. Since horizontal gene transfer can be considered an important exogenous dynamic resulting in cross-species infection of these microorganisms, can endogenous genes, which function as important roles in life cycle of *Chlamydia*, be influenced by host bacteria? *Pmps* genes are considered as a highly heterogenous gene family and possess key biological activities in life cycle of *Chlamydiae*, and *T3ss* effectors which also play important roles in life cycle represent high variation of sequence similarity within *Chlamydiaceae* [1,59,60]. Interestingly, *T3ss* and *Pmps* genes represent strong codon usage adaptation to the corresponding host bacteria, implying that during formation of codon usage pattern, genes with important functions in *Chlamydia* spp. undergo natural selection from host bacteria. Previous reports have pointed out that those genes with important biological activities have a strong codon adaptation to their corresponding genomes, including ribosomal major transcription/translational processing factors, major chaperone/degradation proteins and also genes encoding enzymes of fatty acid biosynthesis, amino acid, and nucleotide biosynthesis [51,61,62].

## 4. Materials and Methods

### 4.1. The Genome Data

14 whole genomes of the 12 chlamydial species (three genomes for *C. trachomatis*) in the *Chlamydiaceae* family with coding sequence annotations were obtained from the National Center for Biotechnology (NCBI) GenBank database. The demographics of the selected species were given in Table S1.

### 4.2. Nucleotide Usage Patterns by Information Entropy

To clarify the effects of nucleotide composition on codon usage patterns, the following compositional properties were calculated for the coding sequences of the 14 genomes, namely the overall frequency of occurrence of nucleotides (N%, ‘N’ meaning any nucleotides), frequency of each nucleotide at the third codon position (N3%) and frequency of occurrence of nucleotides GC at the third codon position (GC3%). According to the pr evious report about analyzing nucleotide usage bias [32,55], we employed information entropy to reflect the overall nucleotide usage bias and nucleotide usage bias at the third codon position.

$$Entropy=-\sum_{i}f_{i}\times \log_{2}(f_{i})$$

$$f_{i}=\frac{F_{i}}{F(A)+F(T)+F(G)+F(C)}$$

where *f_i_* means the probability of the specific nucleotide (*F_i_*), *F_i_* means a number of occurrences of the specific nucleotide. The value of *Entropy* for nucleotide usage bias represents how dispersed the contribution of these four types of nucleotide is: the higher value, the more uniform nucleotide usage is; in contrast, the lower value reflects a more biased usage of nucleotide.

### 4.3. Relative Synonymous Codon Usage (RSCU) Value

The RSCU values for all coding sequences of the 12 chlamydial genomes were calculated to determine the characteristics of synonymous codon usage without the confounding influences of the amino acid usages or the gene *lengths* [63]. It is obvious that RSCU values close to 1.0 indicate a lack of bias for the corresponding codons, in contrast, RSCU values deviating from 1.0 reflect usage bias for the corresponding codons. When the RSCU value is 1.0, the corresponding synonymous codon is selected equally and randomly. Furthermore, to better reflect the extent of synonymous codon usage trends, RSCU values more than 1.6 and less than 0.6 were regarded as ‘over-represented’ and ‘under-represented’ codons, respectively [64].

### 4.4. Amino Acid Usage Bias by Information Entropy

To better clarify the extent of amino acid usage bias, we reference d the formation of nucleotide usage bias mentioned above. As for amino acid usage bias of each gene, the information Entropy over the frequencies of different amino acids in a given gene is represented by the formula [32,55]:

$$Entropy=-\sum_{i}f_{i}\times \log_{2}(f_{i})$$

$$f_{i}=\frac{F_{i}}{\sum_{i=1}^{20}F_{i}}$$

where *f_i_* means the probability of the specific nucleotide (*F_i_*). *F_i_* means a number of occurrences of the specific amino acid. The total types of amino acid are 20. The value of *Entropy* for amino acid usage bias ranges from 0 to 1, and represents how a dispersed contribution of the twenty types of amino acid: the higher value, the more uniform amino acid usage; in the contrast, the lower value reflects the more biased usage of amino acid.

### 4.5. Genetic Diversity of Chlamydia at Synonymous Codon and Amino Acid Usages

Principal component analysis (PCA) is a multivariate statistical method which reduces data dimensionality by performing a covariance analysis for a data matrix. As for genetic diversity for *Chlamydia* at gene levels, PCA was carried out by RSCU data of the 14 genomes of 12 chlamydial species. As for genetic diversity for *Chlamydia* at amino acid levels, PCA was carried out by amino acid compositions of them.

### 4.6. Codon Usage Index

To better identify the relationship between nucleotide usages and the overall codon usage bias, an effective number of codons (ENC) analysis was introduced in this study [65]. The ENC values range from 20 to 61, which are able to reflect the role of GC3 content in the overall codon usage bias. The lower the ENC value, the more biased the overall codon usage. In addition, to identify the relationship between the overall codon usage bias and protein properties, codon adaptation index (CAI), the grand average of hydropholicity scale and aromaticity were used in this study. These codon index data for coding sequences of the 14 *Chlamydia* genomes were calculated by CodonW software (https://sourceforge.net/projects/codonw/).

### 4.7. Similarity of Codon Usage

To better quantify extents of codon usage adaptation of *Pmps* genes or *T3ss* genes to the corresponding species, *R* (*A*,*B*) index was introduced in this study. The formula for *R* (*A*,*B*) index was calculated as follows:

$$R\;(A,B)=\frac{\sum_{i=1}^{5\;9}a_{i}\times b_{i}}{\sqrt{\sum_{i=1}^{5\;9}a_{i}\times \sum_{i=1}^{5\;9}b_{i}}}$$

$$D\;(A,B)=\frac{1-R\;(A,B)}{2}$$

where *R* (*A*,*B*) index is defined as a cosine value of an included angle between *A* and *B* special vectors meaning the degree of similarity between *Pmps*/*T3ss* gene and the specific species at the aspect of the overall codon usage pattern, *a_i_* is defined as the RSCU value for a specific codon in 59 synonymous codons of coding sequence of Pmps or T3SS, *b_i_* is termed as the RSCU value for the same codon of the corresponding species. *D* (*A*,*B*) index represents the potential effect of the overall codon usage of host on that of DENV, and this value ranges from zero to 1.0 [66].

### 4.8. Statistical Methods

One-way ANOVA method was used to compare means of two or more groups containing numerical response data using the software SPSS 16.0 (IBM, Chicago, IL, USA) for Windows, and significant difference can be identified when *p* value <0.05. Correlation analysis was performed to identify the relationships between CAI data and ENC data/between ENC data and GC3 content for each strain using Spearman’s correlation method.

## Figures and Tables

**Table 1:** Nucleotide content (%) of the 14 *Chlamydia* strains at gene levels.

Species	T%	C%	A%	G%	T3%	C3%	A3%	G3%
*C. trachomatis* L2/25567R	29.9 ± 3.18	20 ± 3.37	28.4 ± 3.31	21.7 ± 3.38	36.5 ± 4	16.6 ± 5.05	29.01 ± 3.93	17.9 ± 4.73
*C. trachomatis* A-Har-13	29.9 ± 3.39	20 ± 3.43	28.5 ± 3.49	21.6 ± 3.61	36 ± 4.27	16.6 ± 5.12	29.1 ± 4.15	17.9 ± 4.84
*C. trachomatis* ESW3	29.9 ± 3.2	20 ± 3.37	28.4 ± 3.34	21.7 ± 3.4	29.9 ± 3.2	20 ± 3.37	28.4 ± 3.34	21.7 ± 3.4
*C. suis* MD56	29.5 ± 3.5	20.4 ± 3.5	28.1 ± 3.7	22 ± 3.7	35 ± 4.4	17.5 ± 5.3	28.2 ± 4.3	19.0 ± 5.2
*C. muridarum* Nigg	30.5 ± 3.3	19.5 ± 3.3	28.8 ± 3.5	21.2 ± 3.7	38 ± 4.1	15.6 ± 5.0	29.5 ± 4.2	17.3 ± 5.0
*C. abortus* S26/3	29.9 ± 3.4	19.8 ± 2.8	29.7 ± 3.5	20.6 ± 2.9	36 ± 4.5	17.3 ± 4.4	29.8 ± 4.3	16.8 ± 4.0
*C. psittaci* 6BC	30.2 ± 3.3	19.4 ± 2.6	30.1 ± 3.4	20.3 ± 2.8	38 ± 4.3	16.2 ± 4.3	30.8 ± 4.3	15.5 ± 3.7
*C. pecorum* E58	29.5 ± 3.8	20.3 ± 4.0	28.8 ± 4.1	21.3 ± 3.9	36 ± 5.3	17.3 ± 6.1	28.4 ± 5.3	18.6 ± 5.5
*C. pneumoniae* TW-183	29.5 ± 3.6	20.4 ± 3.2	29.2 ± 3.6	20.9 ± 3.1	36 ± 4.8	18.1 ± 5.1	28.9 ± 4.9	16.9 ± 4.3
*C. gallinacea* 08-12743	31.3 ± 3.7	18.8 ± 3.2	30.3 ± 3.7	19.7 ± 3.5	39 ± 4.8	14.1 ± 4.8	31.6 ± 4.9	15.0 ± 4.6
*C. avium* 10DC88	31.7 ± 3.7	18.2 ± 3.0	30.9 ± 3.7	19.2 ± 3.3	40 ± 5.1	13.2 ± 4.4	32.8 ± 4.9	13.8 ± 4.2
*C. felis* Fe/C-56	30.1 ± 3.3	19.5 ± 2.7	29.9 ± 3.5	20.4 ± 2.9	37 ± 4.4	16.5 ± 4.1	30.4 ± 4.4	16.0 ± 3.9
*C. caviae* GPIC	30.2 ± 3.4	19.3 ± 2.7	30.0 ± 3.5	20.5 ± 2.9	38 ± 4.5	16.0 ± 4.2	30.4 ± 4.5	15.8 ± 3.9
*C. ibidis* 10-1398 6	31.0 ± 3.7	18.5 ± 2.5	30.2 ± 3.6	20.3 ± 3.2	39 ± 4.8	14.5 ± 3.8	30.6 ± 5.0	15.7 ± 3.9

**Table 2:** The correlation analysis for the effective number of codons (ENC) value and GC3 content of *Chlamydia* spp.

Strains	Correlation	Significance
*C. trachomatis* L2/25567R	*r* = 0.369	*p* = 3.7 × 10^−30^
*C. trachomatis* A-Har-13	*r* = 0.512	*p* = 1.8 × 10^−63^
*C. trachomatis* ESW3	*r* = 0.560	*p* = 2.7 × 10^−74^
*C. suis* MD56	*r* = 0.511	*p* = 2.2 × 10^−63^
*C. muridarum* Nigg	*r* = 0.520	*p* = 2.1 × 10^−63^
*C. abortus* S26/3	*r* = 0.590	*p* = 5.3 × 10^−93^
*C. psittaci* 6BC	*r* = 0.585	*p* = 1.0 × 10^−89^
*C. pecorum* E58	*r* = 0.554	*p* = 2.8 × 10^−79^
*C. pneumoniae* TW-183	*r* = 0.488	*p* = 2.8 × 10^−67^
*C. gallinacea* 08-12743	*r* = 0.548	*p* = 1.6 × 10^−71^
*C. avium* 10DC88	*r* = 0.630	*p* = 1.3 × 10^−104^
*C. felis* Fe/C-56	*r* = 0.580	*p* = 4.5 × 10^−89^
*C. caviae* GPIC	*r* = 0.585	*p* = 1.1 × 10^−91^
*C. ibidis* 10-1398/6	*r* = 0.547	*p* = 1.4 × 10^−76^

**Table 3:** The correlation between the codon adaptiation index (CAI) and ENC of *Chlamydia* spp.

Strains	Correlation	Significance
*C. trachomatis* L2/25567R	*r* = 0.272	*p* = 1.6 × 10^−16^
*C. trachomatis* A-Har-13	*r* = −0.186	*p* = 9.6 × 10^−9^
*C. trachomatis* ESW3	*r* = −0.257	*p* = 7.4 × 10^−15^
*C. suis* MD56	*r* = −0.417	*p* = 9.8 × 10^−41^
*C. muridarum* Nigg	*r* = −0.16	*p* = 1.5 × 10^−6^
*C. abortus* S26/3	*r* = −0.299	*p* = 9.8 × 10^−22^
*C. psittaci* 6BC	*r* = −0.311	*p* = 4.9 × 10^−23^
*C. pecorum* E58	*r* = 0.005	*p* = 0.882
*C. pneumoniae* TW-183	*r* = −0.04	*p* = 0.187
*C. gallinacea* 08-12743	*r* = −0.168	*p* = 4.0 × 10^−7^
*C. avium* 10DC88	*r* = −0.308	*p* = 5.4 × 10^−22^
*C. felis* Fe/C-56	*r* = −0.268	*p* = 1.4 × 10^−17^
*C. caviae* GPIC	*r* = −0.275	*p* = 1.4 × 10^−18^
*C. ibidis* 10-1398/6	*r* = −0.246	*p* = 9.1 × 10^−15^

## Supplementary Materials

Supplementary materials can be found at http://www.mdpi.com/1422-0067/19/12/4010/s1.

## Abbreviations

ENC	Effective Number of Codons
CAI	Codon Adaptation Index
RSCU	Relative Synonymous Codon Usage
PCA	Principal Component Analysis
Pmps	Polymorphic membrane proteins
T3ss	Type III secretion system
