# Supplementary figures and images for: Synonymous Codon Usages as an Evolutionary Dynamic for Chlamydiaceae
Source: Int J Mol Sci. 2018 Dec 12;19(12):4010. doi: 10.3390/ijms19124010 (PMC6321445; doi:10.3390/ijms19124010)

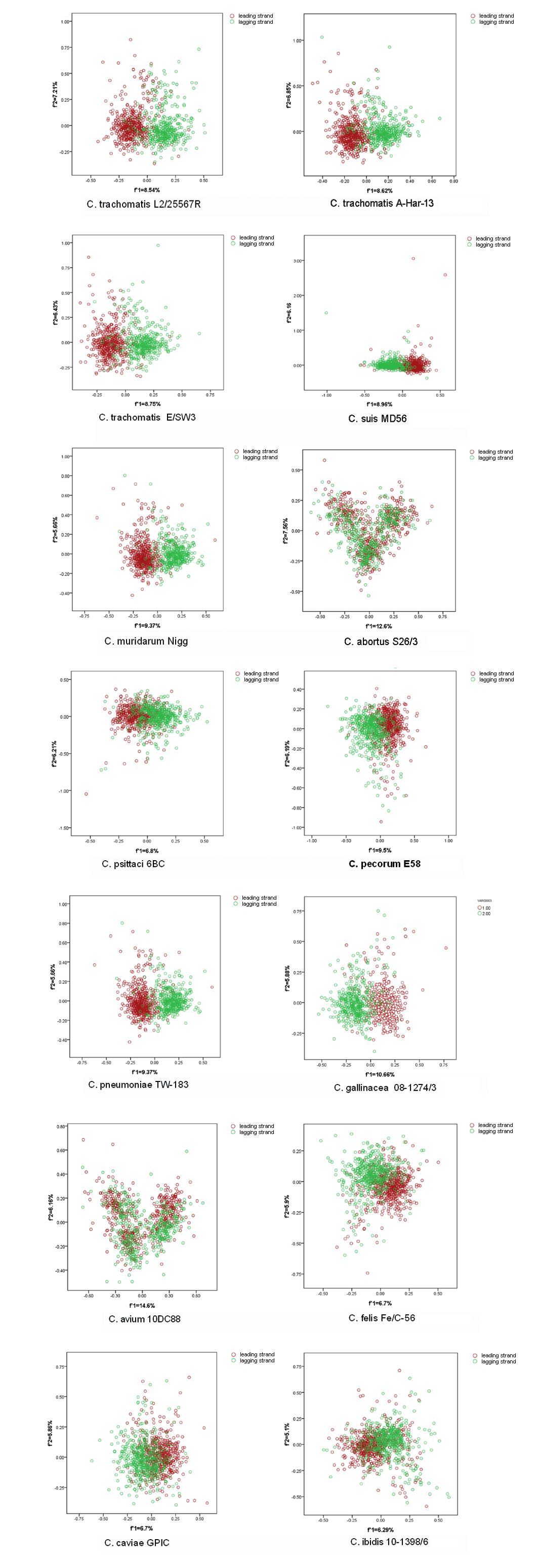

Supplement: Supplementary file 1 [file ijms-19-04010-s001.zip › Figure S1.tif]
