# Supplementary material for: Synonymous Codon Usages as an Evolutionary Dynamic for Chlamydiaceae
Source: Int J Mol Sci. 2018 Dec 12;19(12):4010. doi: 10.3390/ijms19124010 (PMC6321445; doi:10.3390/ijms19124010)
Supplement: Supplementary file 1 [file ijms-19-04010-s001.zip › R1 Table S3 Stop codon usage.doc]

*Table S3 Stop codon usages bias for Chlamydiaceae*

|  | **TAA(*)** | **TAG(*)** | **TGA(*)** |
| --- | --- | --- | --- |
| C. trachomatis L2/25567R | 1.65 | 0.93 | 0.42 |
| C. trachomatis A-Har-13 | 1.47 | 1.00 | 0.52 |
| C. trachomatis ESW3 | 1.65 | 0.91 | 0.44 |
| C. suis MD56 | 1.58 | 0.84 | 0.58 |
| C. muridarum Nigg | 1.74 | 0.82 | 0.44 |
| C. abortus S26/3 | 1.51 | 0.93 | 0.56 |
| C. psittaci 6BC | 1.83 | 0.79 | 0.38 |
| C. pecorum E58 | 1.75 | 0.84 | 0.41 |
| C. pneumoniae TW-183 | 1.64 | 0.88 | 0.47 |
| C. gallinacea 08-12743 | 1.71 | 0.81 | 0.48 |
| C. avium 10DC88 | 1.77 | 0.72 | 0.5 |
| C. felis FeC-56 | 1.69 | 0.87 | 0.44 |
| C. caviae GPIC | 1.9 | 0.75 | 0.35 |
| C. ibidis 10-1398/6 | 1.55 | 0.95 | 0.5 |
