# Supplementary material for: Synonymous Codon Usages as an Evolutionary Dynamic for Chlamydiaceae
Source: Int J Mol Sci. 2018 Dec 12;19(12):4010. doi: 10.3390/ijms19124010 (PMC6321445; doi:10.3390/ijms19124010)
Supplement: Supplementary file 1 [file ijms-19-04010-s001.zip › Table S1.doc]

Table S1. The information about genomes of 12 species in *Chlamydiaceae*

| Species | Accession No. | GC composition | Number of  Coding sequences | Host |
| --- | --- | --- | --- | --- |
| *C. trachomatis* L2/25667R | HE601954 | 41.3% | 941 | Human |
| *C. trachomatis* A-Har-13 | CP000051.1 | 41.3% | 954 | Human |
| *C. trachomatis* E/SW3 | HE601801 | 41.3% | 947 | Human |
| *C. suis* MD56 | AYKJ00000000.1 | 42.0% | 959 | Pig |
| *C. muridarum* Nigg | AE002160 | 40.3% | 946 | Mouse |
| *C. abortus* S26/3 | CR848038.1 | 39.9% | 981 | Sheep |
| *C. psittaci* 6BC | CP002549 | 39.1% | 1024 | Parrot |
| *C. pecorum* E58 | CP002608 | 41.1% | 978 | Calf |
| *C. pneumoniae* TW-183 | AE009440 | 40.6% | 1109 | Human |
| *C. gallinacea* 08-12743 | CP015840 | 37.9% | 951 | Chicken |
| *C. avium* 10DC88 | CP006571 | 36.9% | 890 | Pigeon |
| *C. felis* FeC-56 | NC_007899 | 39.4% | 1011 | Cat |
| *C. caviae* GPIC | AE015925 | 39.2% | 1012 | Guinea pig |
| *C. ibidis* 10-1398/6 | APJW01000001~ APJW01000004 | 38.3% | 1059 | Sacred ibis |
